# Supplementary material for: Non-Coding RNAs as Biomarkers for Embryo Quality and Pregnancy Outcomes: A Systematic Review and Meta-Analysis
Source: Int J Mol Sci. 2023 Mar 17;24(6):5751. doi: 10.3390/ijms24065751 (PMC10052053; doi:10.3390/ijms24065751)
Supplement: Supplementary file 1 [file ijms-24-05751-s001.zip › Table S1.pdf]

Table S1: Search strategy for databases

Database	Search Terms
PubMed (https://www.ncbi.nlm.nih.gov/pubmed) Last search date: 31 July 2022	1. “RNA, Untranslated” [Mesh]
	2. “RNA, Small Untranslated” [Mesh]
	3. “MicroRNAs” [Mesh]
	4. “Circulating MicroRNA” [Mesh]
	5. “non-coding RNA*”
	6. “noncoding RNA*”
	7. ncRNA*
	8. sncRNA*
	9. #1 OR #2 OR #3 OR #4 OR #5 OR #6 OR #7 OR #8
	10. “Embryo Implantation” [Mesh]
	11. “embryo quality”
	12. "Pregnancy Outcome"[Mesh]
	13. #10 OR #11 OR #12
	14. “embryo culture medi*”
	15. “spent culture medi*”
	16. “culture medi*”
	17. “blastocyst culture medi*”
	18. “spent medi*”
	19. “follicular fluid”
	20. “plasma”
	21. #14 OR #15 OR #16 OR #17 OR #18 OR #19 OR #20
	22. #9 AND #13 AND #21
EMBASE via OVID Last search date: 31 July 2022	1. “untranslated RNA”/ or “non-coding RNA*”.tw.
	2. microRNA/ or microRNA*.tw. or “circulating microRNA”/
	3. “long untranslated RNA”/ or “long noncoding RNA*”.tw.
	4. #1 OR #2 OR #3
	5. “embryo quality”.tw.
	6. “embryo implantation”.tw.
	7. “pregnancy outcome”/<br>8. #5 OR #6 OR #7

	9. “culture medium”/ or “embryo culture medi*”.tw.
	10. “follicular fluid”.tw. or “ovary follicle fluid”/
	11. plasma/ or “seminal plasma”/ or plasma.tw.
	12. #9 OR #10 OR #11
	13. #4 AND #8 AND #12
Web of Science (https://www. webofscience. com) Last search date: 31 July 2022	1. ALL= “untranslated RNA”
	2. ALL= “small untranslated RNA”
	3. ALL= microRNA*
	4. ALL= “circulating microRNA*”
	5. ALL= “non-coding RNA*”
	6. ALL= “noncoding RNA*”
	7.ALL= ncRNA*
	8. ALL= sncRNA*
	9. #1 OR #2 OR #3 OR #4 OR #5 OR #6 OR #7 OR #8
	10. ALL= “embryo implantation”
	11. ALL= “embryo quality”
	12. ALL= "pregnancy outcome"
	13. #10 OR #11 OR #12
	14. ALL= “embryo culture medi*”
	15. ALL= “spent culture medi*”
	16. ALL= “culture medi*”
	17. ALL= “blastocyst culture medi*”
	18. ALL= “spent medi*”
	19. ALL= “follicular fluid”
	20. ALL= “plasma”
	21. #14 OR #15 OR #16 OR #17 OR #18 OR #19 OR #20
	22. #9 AND #13 AND #21
