# Supplementary material for: Non-Coding RNAs as Biomarkers for Embryo Quality and Pregnancy Outcomes: A Systematic Review and Meta-Analysis
Source: Int J Mol Sci. 2023 Mar 17;24(6):5751. doi: 10.3390/ijms24065751 (PMC10052053; doi:10.3390/ijms24065751)
Supplement: Supplementary file 1 [file ijms-24-05751-s001.zip › Table S2.pdf]

Table S2. Inclusion and exclusion criteria based on Population, Intervention, Comparator, Outcome, and Study (PICOS) approach.

Parameter	Inclusion	Exclusion
Population	Infertile women undergoing IVF or ICSI	Species other than humans and cell lines
Intervention	Detection of sncRNAs in the extracellular environment	Detection of sncRNAs within cells or tissues
Comparator	Expression of sncRNAs related to pregnancy outcomes or embryo quality:<br>Differential sncRNAs expression between pregnant and non-pregnant embryos<br>Differential sncRNAs expression between aneuploid and euploid embryos<br>Differential sncRNAs expression between embryos of different morphologic grades	Descriptive studies only analyze sncRNAs content in one single population, or studies without comparison
Outcomes	sncRNAs as biomarkers for embryo quality or successful implantation	
Study design	Research article; Comparative study; Cross-sectional study; Retrospective study; Cohort study	Review article; Systematic reviews; Letters; Commentary articles; Conference abstract

ICSI, intracytoplasmic sperm injection; IVF, in vitro fertilization; sncRNAs: small non-coding RNAs.
